# The Association between Nonalcoholic Fatty Liver Disease and CT-Measured Skeletal Muscle Mass

**DOI:** 10.3390/jcm7100310

**Published:** 2018-09-28

**Authors:** Eun Kyung Choe, Hae Yeon Kang, Boram Park, Jong In Yang, Joo Sung Kim

**Affiliations:** 1 Department of Surgery, Healthcare Research Institute, Seoul National University Hospital Healthcare System Gangnam Center, Seoul 06236, Korea; choe523@gmail.com; 2 Department of Internal Medicine, Healthcare Research Institute, Seoul National University Hospital, Healthcare System Gangnam Center, 737 Yeoksam-dong, Gangnam-gu, Seoul 06236, Korea; drmirinae@snuh.org (J.I.Y.); jooskim@snu.ac.kr (J.S.K.); 3 Department of Public Health Science, Seoul National University, Seoul 08826, Korea; 90may11@gmail.com; 4 Department of Internal Medicine, Liver Research Institute, Seoul National University College of Medicine, Seoul 03080, Korea

**Keywords:** nonalcoholic fatty liver disease, sarcopenia, skeletal muscle, CT

## Abstract

A relationship between nonalcoholic fatty liver disease (NAFLD) and sarcopenia has been suggested. The aim of this study was to evaluate the association between NAFLD and skeletal muscle mass measured by computed tomography (CT). The clinical records of individuals visiting our center for a routine health check-up who underwent abdominal ultrasonography and abdominal CT scanning were retrospectively reviewed. Sarcopenia was diagnosed according to body mass index (BMI)-adjusted skeletal muscle mass, which was measured by CT (CT-measured skeletal muscle index (SMI_CT_)). Of the 1828 subjects (1121 males; mean age 54.9 ± 9.5 years), 487 (26.6%) were obese (BMI ≥ 25 kg/m^2^), and 454 (24.8%) had low muscle mass. Sarcopenic subjects had a significantly higher prevalence of NAFLD than nonsarcopenic subjects, regardless of obesity (35.9% vs. 26.8%, *p* = 0.004 in the nonobese group; 76.6% vs. 63.0%, *p* = 0.003 in the obese group). Sarcopenia was significantly associated with the risk of NAFLD (adjusted odds ratio (OR) (95% confidence interval (CI)), 1.51 (1.15–1.99)), and the risk of NAFLD increased with increasing severity of sarcopenia (adjusted OR (95% CI), 1.45 (1.09–1.92) vs. 2.51 (1.16–5.56), mild vs. severe sarcopenia, respectively). When the risk of NAFLD was analyzed according to the SMI_CT_ quartiles, the adjusted OR and 95% CI for the lowest muscle mass quartile compared to the highest were 1.78 (1.17–2.72) in males and 2.39 (1.13–5.37) in females. Low skeletal muscle mass, which was precisely measured by CT, is independently associated with NAFLD, suggesting that sarcopenia is a risk factor for NAFLD.

## 1. Introduction

A progressive decrease in muscle mass is a common body composition change associated with aging, and this change was described as sarcopenia by Rosenberg in 1989 [1]. Many studies have examined the association between sarcopenia and related diseases, such as metabolic syndrome (MS), cardiovascular disease, and the risk of death in the elderly [2,3,4,5,6]. Nonalcoholic fatty liver disease (NAFLD) is the most common liver disease, and it can range from simple steatosis to nonalcoholic steatohepatitis to cirrhosis [7,8,9]. Recent studies have reported a relationship between sarcopenia and NAFLD, and several mechanisms have been suggested [10,11]. The most important pathogenesis connecting sarcopenia and NAFLD is insulin resistance [12,13]. However, Lee et al. reported that sarcopenia is associated with NAFLD independent of obesity and insulin resistance, suggesting that sarcopenia is an independent risk factor for NAFLD [14].

Sarcopenia has been defined in various ways. The European consensus defined computed tomography (CT) scans and magnetic resonance imaging (MRI) as the gold standard for estimating muscle mass [15]. Skeletal muscle area can be objectively measured on cross-sectional imaging and has been shown to be a valid surrogate for whole-body muscle mass [16,17,18]. However, most previous studies that have reported the relationship between NAFLD and sarcopenia used dual energy X-ray absorptiometry (DXA) or bioelectric impedance analysis (BIA) to measure muscle mass [10,14,16]. Therefore, the aim of this study was to evaluate the association between NAFLD and muscle mass, which was precisely measured by CT.

## 2. Patients and Methods

### 2.1. Study Population

We performed a retrospective, cross-sectional study. The clinical records of 3069 subjects who underwent blood sampling, abdominal ultrasonography, and abdominal CT scanning during routine health check-ups between January 2009 and December 2014 at the Seoul National University Hospital Healthcare System Gangnam Center were reviewed. We excluded 1241 subjects with a positive serologic marker for hepatitis B surface antigen or hepatitis C virus serological marker, excessive alcohol intake (>30 g/day for males and >20 g/day for females), other specific hepatic diseases, or a history of malignant disease. Ultimately, 1828 subjects were enrolled in this study. The study protocol was approved by the Institutional Review Board of Seoul National University Hospital (H-1606-095-771), and the requirement for informed consent was waived.

### 2.2. Clinical and Laboratory Assessments

Each subject answered a questionnaire on their medical history and completed an anthropometric assessment and laboratory tests on the same day. Height and body weight were measured using a digital scale. BMI (kg/m^2^) was calculated as the weight divided by the height squared, and waist circumference (WC) was measured at the midpoint between the lower costal margin and the iliac crest by a well-trained nurse. Systolic and diastolic blood pressure (BP) were measured twice, and the mean values were recorded.

The laboratory evaluation included the levels of alanine aminotransferase (ALT), aspartate aminotransferase (AST), gamma-glutamyl transpeptidase (GGT), total cholesterol (TC), triglycerides (TG), low-density lipoprotein (LDL) cholesterol, high-density lipoprotein (HDL) cholesterol, fasting glucose, hemoglobin A1c (HbA1c), hepatitis B surface antigens, and antibodies to the hepatitis C virus. Venous blood samples were collected before 10 a.m. after a 12 h overnight fast.

The subjects were examined in the supine position with a 16-detector row CT scanner (Somatom Sensation 16; Siemens Medical Solutions, Forchheim, Germany). The skeletal muscle area was measured as in previous studies [16,19,20]. The third lumbar vertebrae (L3) was selected as a standard landmark; the L3 region contains the psoas, paraspinal, and abdominal wall muscles. We used a CT software program (Rapidia 2.8; INFINITT, Seoul, Korea) that electronically determines the skeletal muscle area by setting the attenuation values for a region of interest within a range of −29 to 150 Hounsfield units, as previously described [16,19]. A trained technician corrected the boundary of the entire L3 skeletal muscle area twice, and the average value was used for analysis. This value was normalized for BMI (kg/m^2^), according to the guidelines of the Foundation for the National Institutes of Health (NIH) Sarcopenia Project [17] and was reported as the CT-measured skeletal muscle index (SMI_CT_) (cm^2^/(kg/m^2^)).

### 2.3. Definitions

Smoking status was self-reported as never, ex- and current. Diabetes mellitus was defined as the current use of anti-diabetic drugs or a fasting glucose level of 126 mg/dL or higher. Hypertension was defined as the current use of anti-hypertensive drugs, a systolic BP greater than 140 mmHg, or a diastolic BP greater than 90 mmHg. MS was diagnosed when three or more of the following five components were present, based on the modified National Cholesterol Education Program Adult Treatment Panel III [18]: (1) central obesity [defined as a WC > 90 cm (men) or > 80 (women), according to the Regional Office for the Western Pacific Region of the World Health Organization criteria]; (2) triglyceride levels ≥150 mg/dL; (3) HDL cholesterol levels <40 mg/dL (men) or <50 mg/dL (women); (4) fasting glucose levels ≥100 mg/dL or the use of anti-diabetic medications; (5) BP ≥ 130/85 mmHg or the use of anti-hypertensive medications.

NAFLD was defined as the presence of a fatty liver on ultrasonography in the absence other possible causes of chronic liver diseases. Fatty liver was diagnosed by ultrasonographic findings (Acuson, Sequoia 512, Siemens, Mountain View, CA, USA) based on liver brightness, echo contrast between the hepatic and renal parenchyma, vascular burring, and deep attenuation [21,22]. Ultrasonographic examination of the liver was performed by experienced radiologists blinded to the subjects’ laboratory and clinical data.

Sarcopenia was defined as SMI_CT_ 1 standard deviation (SD) below the sex-specific mean value for the young healthy population (18–40 years). By this definition, the cut-off values were 8.37 cm^2^/(kg/m^2^) for males and 7.47 cm^2^/(kg/m^2^) for females. The cut-off points for sarcopenia were 20.2% for males and 32.3% for females (*p* < 0.001). We grouped the participants further according to the severity of sarcopenia. Mild sarcopenia was indicated in participants whose SMI_CT_ was between 1 and 2 SD below the mean. Severe sarcopenia was indicated in participants whose SMI_CT_ was 2 SD below the mean (7.04 cm^2^/(kg/m^2^) for males and 6.12 cm^2^/(kg/m^2^) for females) [23]. Obesity was defined as a BMI greater than or equal to 25 kg/m^2^ according to the BMI categories for Asians recommended by the International Obesity Task Force (WHO Western Pacific Region, 2000) [24,25]. Sarcopenic obesity was defined as the combination of sarcopenia and obesity according to International Obesity Task Force definitions.

### 2.4. Statistical Analysis

Comparisons of continuous variables between the two groups were performed using Student’s *t*-test, and categorical variables were compared using the chi-square test or Fisher’s exact test. Standard muscle mass quartiles were categorized separately by sex as follows: for males, quartile (Q) 1: ≤8.53; Q2: 8.53–9.25; Q3: 9.25–10.16; and Q4: >10.16 cm^2^/(kg/m^2^); for females, Q1: ≤7.22; Q2: 7.22–7.93; Q3: 7.93–8.94; and Q4: >8.94 cm^2^/(kg/m^2^). Variables that were statistically significant in the univariate analysis and are known risk factors were included in a multiple logistic regression model to identify the independent predictors of NAFLD and sarcopenia. To estimate the *p* for the trend, the Cochran–Armitage test for trends was performed.

Statistical analyses were performed using the Statistical Package for the Social Sciences, version 22.0 (SPSS, Inc., Chicago, IL, USA), and R statistical software, version 3.2.2 (R Development Core Team; R Foundation for Statistical Computing, Vienna, Austria). Statistical significance was established for two-sided *p* values <0.05.

## 3. Results

A total of 1828 subjects (1121 males, mean age 54.9 ± 9.5 years) were included for analysis in this study. Of the study subjects, 487 (26.6%) were obese, and 454 (24.8%) had low muscle mass. The baseline characteristics of the study population classified according to BMI and muscle mass are shown in Table 1. The prevalence of sarcopenia was higher in the obese group than in the nonobese group (20.1% for the nonobese group vs. 37.8% for the obese group; *p* < 0.001). Both the obese and nonobese sarcopenic subjects were older, had higher BMIs, were more likely to be female, and were less likely to be smokers than the nonsarcopenic subjects.

**Figure 1:**
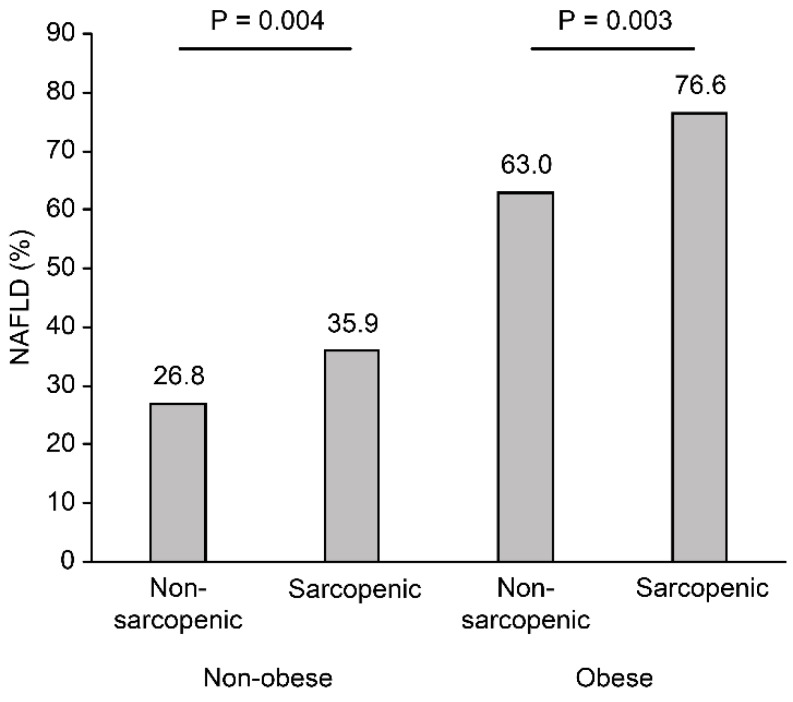
The proportion of the subjects with nonalcoholic fatty liver disease (NAFLD) according to sarcopenia status, stratified by body mass index (BMI). The occurrence of NAFLD was significantly higher in sarcopenic subjects than in nonsarcopenic subjects stratified by BMI.

The MS factor differed significantly different between the sarcopenic and nonsarcopenic subjects in the nonobese group, but not in the obese group. In the nonobese group, systolic BP, WC, TC, TG, and LDL cholesterol were higher, and HDL cholesterol was lower, in sarcopenic subjects than in nonsarcopenic subjects (all *p* < 0.05). However, HDL cholesterol was higher in sarcopenic subjects than in nonsarcopenic subjects, and the other metabolic factors were not significantly different between the sarcopenic and nonsarcopenic subjects in the obese group. The sarcopenic subjects had a significantly higher prevalence of NAFLD than the nonsarcopenic subjects, regardless of obesity (35.9 vs. 26.8%, *p* = 0.004 in the nonobese group; 76.6% vs. 63%, *p* = 0.003 in the obese group) (Figure 1).

The mean SMI_CT_ values for the subjects with and without NAFLD are shown in Table 2. The mean SMI_CT_ values were significantly lower in the subjects with NAFLD than in those without NAFLD (8.64 ± 1.29 vs. 9.10 ± 1.51, respectively, *p* < 0.001), and this difference remained significant after adjusting for multiple variables such as age, sex, WC, systolic BP, fasting glucose, TG, HDL cholesterol, and smoking (8.65 ± 0.90 vs. 9.11 ± 0.89, respectively, *p* < 0.001).

We analyzed the association between sarcopenia and NAFLD. The risk of NAFLD increased according to the severity of sarcopenia (age- and sex-adjusted odds ratio (OR) (95% confidence interval (CI)) 2.50 (1.96–3.19) vs. 4.88 (2.47–9.93), mild vs. severe sarcopenia, respectively, as shown in Table 3). This effect of sarcopenia remained significant in the multivariate analysis, in which other well-identified risk factors for NAFLD were considered. Sarcopenia was significantly associated with the risk of NAFLD (adjusted OR (95% CI) 1.51 (1.15–1.99)). The risk of NAFLD increased according to the severity of sarcopenia after multiple variables were adjusted (adjusted OR (95% CI), 1.45 (1.09–1.92) vs. 2.51 (1.16–5.56), mild vs. severe sarcopenia, respectively).

**Figure 2:**
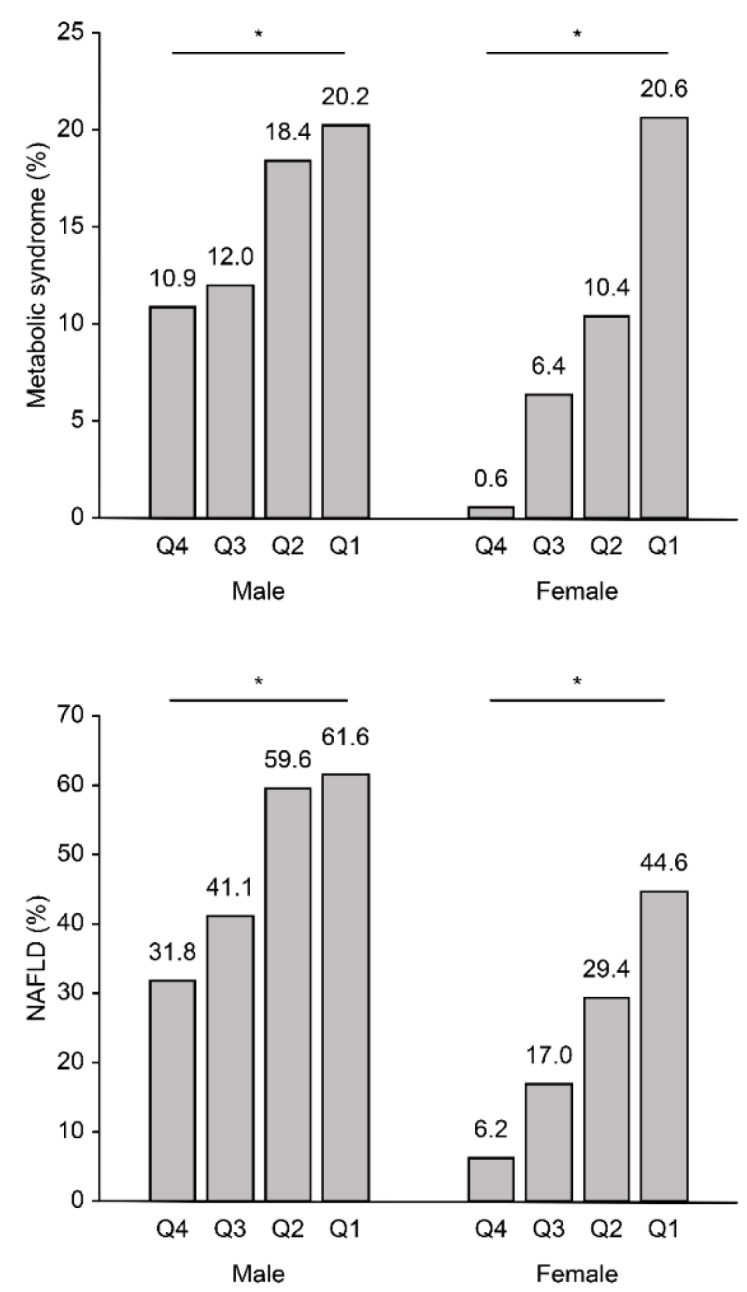
The proportion of the subjects with metabolic syndrome (MS) and nonalcoholic fatty liver disease (NAFLD) according to the computed tomography (CT)-measured skeletal muscle index (SMI_CT_) quartiles in males and females. * *p*-value for the test of the trend of the odds <0.001. For males, quartile (Q) 1: ≤8.53; Q2: 8.53–9.25; Q3: 9.25–10.16; and Q4: >10.16 cm^2^/(kg/m^2^); for females, Q1: ≤7.22; Q2: 7.22–7.93; Q3: 7.93–8.94; and Q 4: >8.94 cm^2^/(kg/m^2^).

We also analyzed the risk of NAFLD according to the SMI_CT_ quartiles (Figure 2, Table 4). Figure 2 shows the proportion of the subjects with MS and NAFLD according to the muscle mass quartiles. The percentage of subjects with MS gradually increased as the muscle mass quartile decreased (10.9%, 12.0%, 18.4%, and 20.2% in males and 0.6%, 6.4%, 10.4%, and 20.6% in females in Q4, Q3, Q2, and Q1, respectively; *p* for trend <0.001 in males and females). The percentage of subjects with NAFLD gradually increased as the SMI_CT_ quartile decreased (31.8%, 41.1%, 59.6%, and 61.6% in males and 6.2%, 17.0%, 29.4%, and 44.6% in females in Q4, Q3, Q2, and Q1, respectively; *p* for trend <0.001 in males and females). Table 4 shows the risk of NAFLD according to the SMI_CT_ quartiles. After adjusting for multiple variables, the risk of NAFLD significantly increased as the SMI_CT_ decreased (*p* for trend <0.001 in males and females). In the multiple logistic regression analysis, the OR for NAFLD risk was 1.78 (1.17–2.72) in males and 2.39 (1.13–5.37) in females in the lowest quartile of SMI_CT_ compared to the risk in the highest quartile after adjusting for potential confounding factors.

## 4. Discussion

To the best of our knowledge, this is the first study on the relationship between NAFLD and muscle mass measured by CT scanning. In this study, low muscle mass, which was precisely measured by CT, was found to be independently associated with NAFLD, suggesting that sarcopenia is a risk factor for NAFLD. The prevalence of NAFLD was significantly higher in sarcopenic subjects than in nonsarcopenic subjects, regardless of obesity. In addition, the risk for NAFLD increased according to the severity of sarcopenia. When we analyzed the risk of MS and NAFLD according to the standard muscle quartiles, the subjects with lower muscle mass exhibited an increased risk of MS and NAFLD in the multivariate analysis. These results indicate that NAFLD is proportionally affected by the amount of muscle, even if the muscle does not decrease at the sarcopenia level.

Various methods have been developed to measure skeletal muscle mass to determine the sarcopenia status. In most previous studies, muscle mass was measured by DXA or BIA [10,14,26]. However, CT and MRI are considered the gold standards for estimating muscle mass, although CT is limited by its relatively high radiation exposure [15,27]. In addition, tests for gait speed and handgrip strength to see muscle function have not been routinely performed. Therefore, the diagnosis of sarcopenia has not been made properly compared to that NAFLD, which has a high global prevalence and is easily detected by ultrasonography or CT [28]. Because sarcopenia has become increasingly significant in the elderly population, it is necessary to identify the best imaging study for determining skeletal muscle mass that can be easily used in clinical practice, and we should consider more aggressive testing for muscle function [28].

In this paper, we used the cross-sectional skeletal muscle area at the level of L3. This value is known to be linearly related to whole-body muscle mass [26]. The muscle mass should be adjusted before determining the sarcopenia status because interpretation using muscle mass alone cannot rule out the effect of body size [26]. Standardization has been performed using various body size measures (i.e., height^2^, weight, BMI, and total body fat) [17,29,30]. Recently, Peng et al. noted that, when muscle mass is adjusted by body weight or BMI, more subjects who are identified as having sarcopenia are overweight or obese [31]. This tendency was also found in our study; a higher BMI and WC were found in the sarcopenic subjects than in the nonsarcopenic subjects in both the obese and nonobese groups. However, adjusting for height tended to underestimate the prevalence of sarcopenia, especially in females [32,33]. In addition, the BMI-adjusted definition was selected as a good predictor of sarcopenia by the FNIH Sarcopenia Project because of its significant association with mobility impairment [17,29,34]. Therefore, we used the CT-measured cross-sectional skeletal muscle area divided by BMI (SMI_CT_) (cm^2^/(kg/m^2^)) for the analysis in this study. There is no cut-off value for sarcopenia measured by CT in the Asian population, so we used the sex-specific mean value of the young healthy population as the cut-off value in the definition of sarcopenia. In addition, the significant relationship between sarcopenia and NAFLD persisted even after adjusting for the components of MS, including WC, which represented central obesity. These findings agree with those of previous studies reporting that sarcopenia is associated with an increased risk of NAFLD, independent of obesity or MS [14,35].

Several possible mechanisms connecting sarcopenia and NAFLD have been suggested. Insulin resistance is a major pathophysiology of sarcopenia and NAFLD because both liver and muscle are target organs for insulin [12,36]. The loss of muscle mass contributes to glucose intolerance and promotes gluconeogenesis by reducing the quantity of the main cellular target for insulin. When insulin resistance occurs in myocytes, muscle mass is depleted by the reduction in protein synthesis and the increase in protein catabolism [28,37]. Therefore, insulin resistance and sarcopenia become a vicious cycle.

Sarcopenia and NAFLD could be related through oxidative stress that occurs as the result of chronic low-grade inflammation [10,38]. Increased levels of inflammatory mediators, such as TNF-α, could contribute to the development of NAFLD by promoting lipid accumulation and could enhance the catabolism of muscle through a reduction in muscle protein synthesis [28,39]. In addition, decreased physical activity and vitamin D deficiency are reported as risk factors for NAFLD and sarcopenia [40,41,42]. A recent meta-analysis by Wijarnpreecha et al. suggested that NAFLD and sarcopenia may not have a causal relationship but could be a result of the same underlying factors [43]. More research is needed on the mechanism underlying the relationship between NAFLD and sarcopenia.

The present study had several strengths compared with previous studies. First, we directly measured the muscle mass of a relatively large number of subjects using CT scanning. Second, this study confirmed the independent association between low muscle mass and NAFLD after adjusting for multiple variables. The risk of NAFLD was stratified according to the amount of skeletal muscle mass adjusted for BMI. Third, we included only relatively homogeneous healthy subjects who visited our center for routine health screening.

However, there were several limitations in this study. First, this study was retrospective and cross-sectional in design; thus, it was difficult to identify causal or temporal relationships between muscle mass and NAFLD. In addition, due to the limitations of retrospective studies, some possible confounding factors, such as vitamin D or chronic inflammation that could affect the pathophysiology of NAFLD and sarcopenia were not considered. However, key factors associated with insulin resistance and the information obtained from the detailed questionnaire were included in this study. Second, this was a single-center study, and the subjects were self-recruited via routine health check-ups. Therefore, our findings might not represent the general population. Third, this study was based on only muscle mass, and the effect of muscle function on NAFLD was not investigated. Fourth, we diagnosed NAFLD with ultrasonographic examination, although liver biopsy is considered the gold standard for the diagnosis of NAFLD [32]. However, liver biopsy has a risk of complications and is therefore difficult to use during a routine health check-up.

In conclusion, this study shows that low muscle mass, which was precisely measured with CT, is significantly associated with NAFLD, independent of other metabolic risk factors. Well-designed longitudinal studies are warranted to assess the progression of NAFLD with changes in muscle mass. Further study is also needed to identify the effects of not only muscle mass but also muscle function on NAFLD.

## Figures and Tables

**Table 1:** The baseline characteristics between subjects with and without sarcopenia.

Variable	Nonobese (*n* = 1341)			Obese (*n* = 487)		
	Nonsarcopenic (*n* = 1071)	Sarcopenic (*n* = 270)	*p* Value	Nonsarcopenic (*n* = 303)	Sarcopenic (*n* = 184)	*p* Value
Age, years	54.0 ± 9.6	58.2 ± 8.9	<0.001	53.5 ± 9.0	58.1 ± 9.2	<0.001
Male, %	610 (57.0)	91 (33.7)	<0.001	285 (94.1)	135 (73.4)	<0.001
Smoking status			<0.001			<0.001
Never	604 (56.8)	213 (80.1)		80 (26.5)	84 (46.2)	
Ex-smoker	343 (32.2)	42 (15.8)		164 (54.3)	84 (46.2)	
Current smoker	117 (11.0)	11 (4.1)		58 (19.2)	14 (7.7)	
Diabetes mellitus	29 (2.7)	6 (2.2)	0.815	14 (4.6)	10 (5.4)	0.852
Hypertension	327 (30.7)	103 (38.4)	0.018	189 (62.6)	111 (60.7)	0.744
Body mass index, kg/m^2^	21.9 ± 1.9	22.7 ± 1.5	<0.001	26.6 ± 1.5	27.0 ± 1.8	0.013
Waist circumference, cm	80.6 ± 6.4	82.9 ± 5.3	<0.001	92.3 ± 5.0	93.0 ± 5.6	0.114
Systolic blood pressure, mmHg	113.5 ± 13.5	116.0 ± 13.9	0.007	121.9 ± 12.8	122.6 ± 11.9	0.548
Diastolic blood pressure, mmHg	73.1 ± 10.2	73.6 ± 10.2	0.514	80.5 ± 10.5	79.0 ± 9.5	0.108
Total cholesterol, mg/dL	191.3 ± 33.7	200.0 ± 36.9	<0.001	190.1 ± 36.8	193.2 ± 34.7	0.352
Triglycerides, mg/dL	95.7 ± 55.4	109.0 ± 65.3	0.002	143.1 ± 86.8	134.7 ± 88.2	0.308
HDL cholesterol, mg/dL	56.1 ± 13.1	54.0 ± 11.4	0.009	46.5 ± 10.3	49.5 ± 11.1	0.003
LDL cholesterol, mg/dL	117.2 ± 30.0	125.0 ± 30.6	0.001	121.6 ± 29.8	124.0 ± 33.9	0.480
AST, IU/L	22.7 ± 8.2	22.8 ± 7.9	0.841	25.4 ± 19.3	25.4 ± 8.6	1.000
ALT, IU/L	21.8 ± 12.4	22.3 ± 14.7	0.591	29.8 ± 23.0	30.7 ± 16.3	0.613
GGT, IU/L	27.1 ± 23.8	27.2 ± 19.1	0.979	43.4 ± 39.6	42.5 ± 36.1	0.796
Fasting glucose, mg/dL	96.0 ± 16.2	96.8 ± 13.2	0.392	104.0 ± 19.7	102.0 ± 14.2	0.204
HbA1c, %	5.7 ± 0.5	5.7 ± 0.4	0.472	5.8 ± 0.6	5.9 ± 0.5	0.330
Muscle mass, cm^2^	208.3 ± 31.5	163.7 ± 20.3	<0.001	247.1 ± 23.4	201.0 ± 24.5	<0.001
SMI_CT_, cm^2^/(kg/m^2^)	9.5 ± 1.3	7.2 ± 0.7	<0.001	9.3 ± 0.8	7.4 ± 0.7	<0.001
Metabolic syndrome	72 (6.8)	32 (12.1)	0.006	80 (26.7)	52 (28.7)	0.700

Data are presented as the mean ± standard deviation or as percentages (%). SMI_CT_: CT-measured skeletal muscle index; HDL: high-density lipoprotein; LDL: low-density lipoprotein; AST: aspartate aminotransferase, ALT: alanine aminotransferase; GGT: gamma-glutamyl transpeptidase; HbA1c: hemoglobin A1c.

**Table 2:** Computed tomography (CT)-measured skeletal muscle index (SMI_CT_) between the control and nonalcoholic fatty liver disease (NAFLD) groups.

Variable	NAFLD (−)	NAFLD (+)	*p* Value
SMI_CT_	9.10 ± 0.05	8.64 ± 0.05	<0.001
SMI_CT_, adjusted model 1	9.10 ± 0.02	8.64 ± 0.03	<0.001
SMI_CT_, adjusted model 2	9.11 ± 0.03	8.64 ± 0.03	<0.001
SMI_CT_, adjusted model 3	9.11 ± 0.03	8.65 ± 0.03	<0.001

The data are presented as the mean ± standard error. Model 1 was adjusted for age and sex. Model 2 was adjusted for age, sex, waist circumference, systolic blood pressure, and fasting glucose. Model 3 was adjusted for age, sex, waist circumference, systolic blood pressure, fasting glucose, triglycerides, high-density lipoprotein (HDL) cholesterol, and smoking status. SMI_CT_: CT-measured skeletal muscle index. NAFLD: nonalcoholic fatty liver disease.

**Table 3:** Multivariate analyses of the risk of nonalcoholic fatty liver disease (NAFLD) in subjects with and without sarcopenia.

Variable	NAFLD (−)	NAFLD (+)	Model 1		Model 2		Model 3	
			OR (95% CI)	*p* Value	OR (95% CI)	*p* Value	OR (95% CI)	*p* Value
No sarcopenia	896	478	1		1		1	
Sarcopenia	216	238	2.63 (2.08–3.34)	<0.001	1.61 (1.24–2.09)	<0.001	1.51 (1.15–1.99)	0.003
No sarcopenia	896	478	1	<0.001 *	1	<0.001 *	1	0.001 *
Mild sarcopenia	201	214	2.50 (1.96–3.19)	<0.001	1.54 (1.18–2.02)	0.002	1.45 (1.09–1.92)	0.010
Severe sarcopenia	15	24	4.88 (2.47–9.93)	<0.001	2.80 (1.30–6.17)	0.009	2.51 (1.16–5.56)	0.020

NAFLD: nonalcoholic fatty liver disease. OR: odds ratio; CI: confidence interval. Model 1 was adjusted for age and sex. Model 2 was adjusted for age, sex, waist circumference, systolic blood pressure, and fasting glucose. Model 3 was adjusted for age, sex, waist circumference, systolic blood pressure, fasting glucose, triglycerides, high-density lipoprotein (HDL) cholesterol, and smoking status. * *p*-value for the test of the trend of the odds.

**Table 4:** Multivariate analysis of the risk of nonalcoholic fatty liver disease (NAFLD) according to the CT-measured skeletal muscle index (SMI_CT_) (cm^2^/(kg/m^2^)) quartiles.

Variable	Q4	Q3	Q2	Q1	*p* for Trend
Male					
Model 1	1 (reference)	1.53 (1.08–2.17)	3.28 (2.32–4.66)	3.70 (2.60–5.31)	<0.001
Model 2	1 (reference)	1.18 (0.81–1.72)	2.08 (1.42–3.06)	1.94 (1.30–2.91)	<0.001
Model 3	1 (reference)	1.09 (0.74–1.61)	1.99 (1.33–2.98)	1.78 (1.17–2.72)	<0.001
Female					
Model 1	1 (reference)	3.01 (1.49–6.51)	5.64 (2.91–11.85)	10.25 (5.36–21.37)	<0.001
Model 2	1 (reference)	1.58 (0.74–3.60)	2.29 (1.11–5.06)	3.06 (1.48–6.75)	<0.001
Model 3	1 (reference)	1.61 (0.73–3.71)	1.76 (0.83–3.98)	2.39 (1.13–5.37)	0.025

Model 1 was adjusted for age. Model 2 was adjusted for age, waist circumference, systolic blood pressure, and fasting glucose. Model 3 was adjusted for age, waist circumference, systolic blood pressure, fasting glucose, triglycerides, high-density lipoprotein (HDL) cholesterol, and smoking status. For males, quartile (Q) 1: ≤8.53; Q2: 8.53–9.25; Q3: 9.25–10.16; and Q4: >10.16 cm^2^/(kg/m^2^); for females, Q1: ≤7.22; Q2: 7.22–7.93; Q3: 7.93–8.94; and Q4: >8.94 cm^2^/(kg/m^2^).
